# Recent increase in catastrophic tropical cyclone flooding in coastal North Carolina, USA: Long-term observations suggest a regime shift

**DOI:** 10.1038/s41598-019-46928-9

**Published:** 2019-07-23

**Authors:** Hans W. Paerl, Nathan S. Hall, Alexandria G. Hounshell, Richard A. Luettich, Karen L. Rossignol, Christopher L. Osburn, Jerad Bales

**Affiliations:** 1 Institute of Marine Sciences, University of North Carolina at Chapel Hill, Morehead City, NC 28557 USA; 2 Department of Marine, Earth and Atmospheric Sciences, North Carolina State University, Raleigh, NC 27607 USA; 3 Consortium of Universities for the Advancement of Hydrologic Science, Cambridge, MA 02140 USA

**Keywords:** Carbon cycle, Climate sciences, Environmental impact

## Abstract

Coastal North Carolina, USA, has experienced three extreme tropical cyclone-driven flood events since 1999, causing catastrophic human impacts from flooding and leading to major alterations of water quality, biogeochemistry, and ecological conditions. The apparent increased frequency and magnitudes of such events led us to question whether this is just coincidence or whether we are witnessing a regime shift in tropical cyclone flooding and associated ecosystem impacts. Examination of continuous rainfall records for coastal NC since 1898 reveals a period of unprecedentedly high precipitation since the late-1990’s, and a trend toward increasingly high precipitation associated with tropical cyclones over the last 120 years. We posit that this trend, which is consistent with observations elsewhere, represents a recent regime shift with major ramifications for hydrology, carbon and nutrient cycling, water and habitat quality and resourcefulness of Mid-Atlantic and possibly other USA coastal regions.

## Introduction

**Figure 1:**
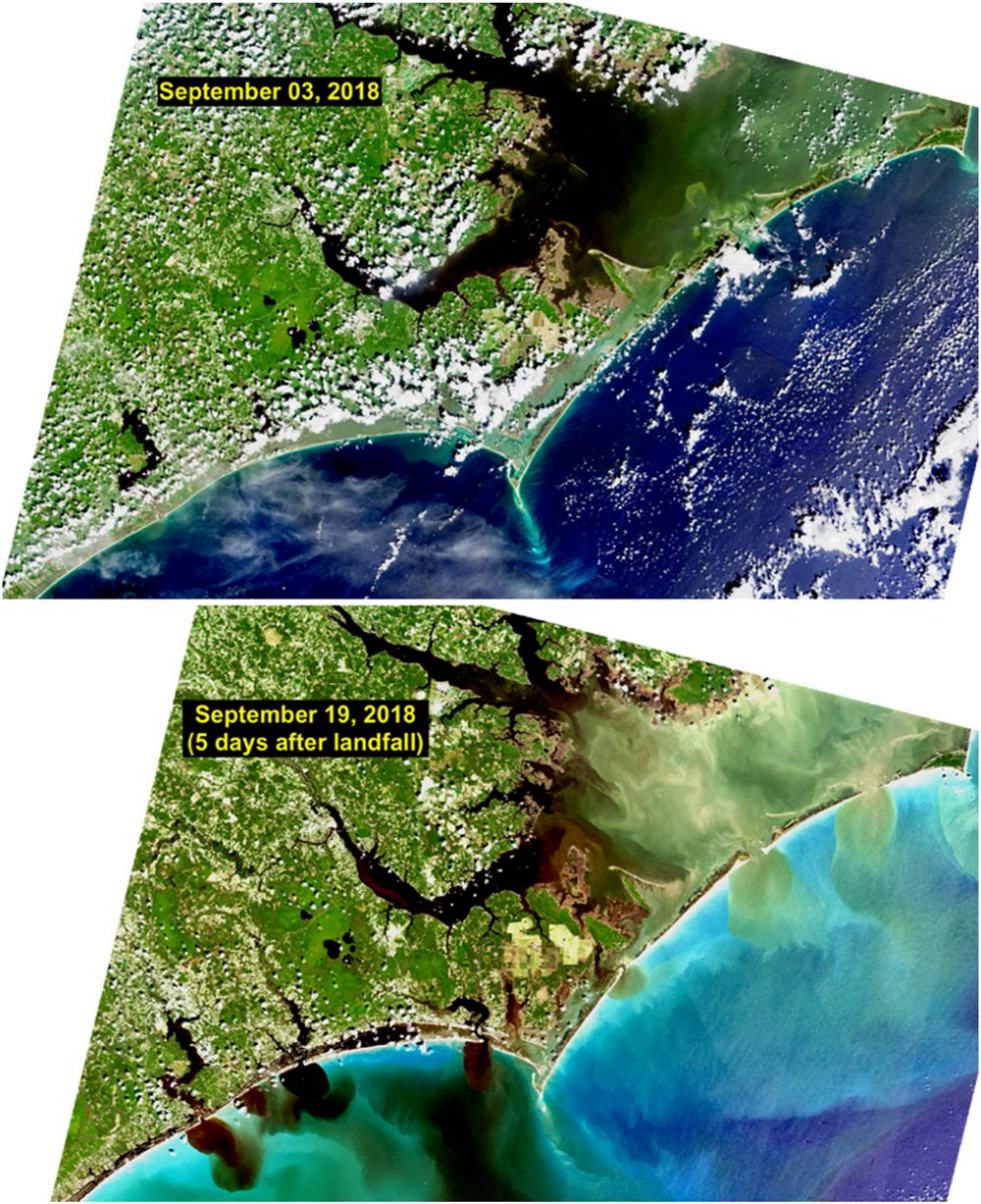
NASA/USGS Landsat images of coastal North Carolina centered on the Neuse River Estuary and lower Pamlico Sound. Top figure shows the system prior to passage of Hurricane Florence on 15 September, 2018. Bottom image shows the same region after the storm, highlighting the export of colored dissolved organic matter (CDOM) from land into coastal waters. Image courtesy NASA and the US Geological Survey.

Since the late-1990’s, coastal North Carolina (NC), USA has been impacted by 36 tropical cyclones, with three recent storms resulting in 2-percent or less annual exceedance levels flood events in the NC coastal plain: At Kinston, NC, Hurricane Floyd (1999) was rated a 2-percent exceedance level flood^1^. Based on the rating scale used to assess flooding from Floyd, the most recent Hurricane Florence (2018) also resulted in a 2-percent annual exceedance level year flood^1^. Peak flow following Hurricane Matthew (2016) resulted in a 0.8-percent annual exceedance level flood in Kinston, NC^2^. This recent apparent increased frequency of extreme events has led us to question whether this is just a coincidence or whether it may reflect the predicted^2,3^ increased frequency of extreme precipitation in a warming climate^4–6^. In addition to their devastating societal and economic impacts, storms associated with this increased frequency are having major ramifications for carbon and nutrient cycling in coastal estuaries and thus represent “hot moments” in coastal biogeochemistry^7^. In fact, recent work shows that these extreme events caused unprecedented nutrient- and organic matter-laden freshwater discharges to nutrient-sensitive receiving coastal waters, including the USA’s 2^nd^ largest estuarine complex and a key fishery and recreational resource, the Albemarle-Pamlico Sound (APS) (Fig. 1), which drains ~ 40% of North Carolina’s and 10% of Virginia’s coastal plain regions via 5 major rivers^8,9^.

In order to quantitatively assess the hydrologic and biogeochemical impacts of the recent rise in these extreme events, we examined freshwater discharge, nutrient (focusing on the algal growth-limiting nutrient, nitrogen) and organic carbon inputs as well as water column salinity, pH and dissolved oxygen associated with tropical cyclones that impacted a key sub-estuary of the APS, the Neuse River Estuary (NRE), as well as western Pamlico Sound (PS), using a 25 year long space-time intensive water quality monitoring program, ModMon^10^. Results were evaluated in the context of a 120 year record of frequency and intensity of tropical cyclones and the rainfall each delivered to this region. Overall, our analysis indicates that; 1) we are experiencing a regime shift in the intensity and quantity of rainfall associated with these events, and 2) this shift has led to unprecedented large loads of nutrients and orgenic matter with major implications for biogeochemical cycling, primary production and overall water quality conditions in the receiving APS and adjacent coastal waters. Furthermore, our observations are consistent with similar observations elsewhere and with predicted hydrologic, nutrient and carbon flux changes taking place in a warming climate^1–6^.

## Materials and Methods

### Study site: neuse river estuary-pamlico sound (NRE-PS)

**Figure 2:**
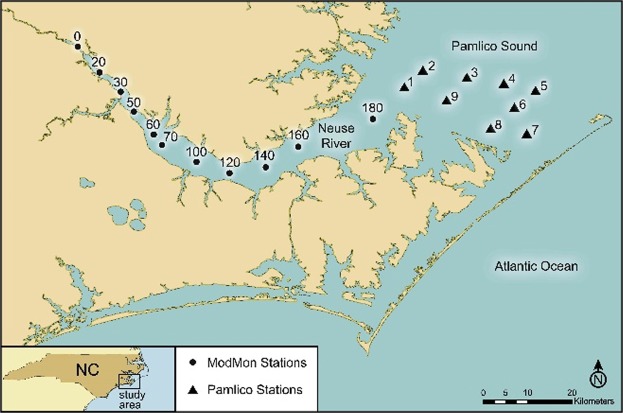
Locations of ModMon Neuse River Estuary and western Pamlico Sound sampling stations and the location of greater Pamlico Sound estuarine system in coastal North Carolina.

The NRE is the second largest tributary of APS in terms of freshwater discharge (Figs 1 and 2). Its watershed is comprised of managed forests and rapidly expanding animal and row-crop agricultural operations. Upstream, the Raleigh-Durham-Research Triangle area has experienced rapid population growth. Nutrient (nitrogen and phosphorus) inputs are dominated by non-point sources (∼80%) associated with these expanding human activities. This has led to accelerating eutrophication, including nuisance algal blooms, hypoxia, and food web changes^11,12^. Changes in the landscape fueling eutrophication are also reflected in organic matter loading to NRE^13^.

The receiving waters of the APS have a surface area of 5,335 km^2^ and drain five major watersheds (Neuse, Tar-Pamlico, Roanoke, Chowan, and Pasquotank Rivers). These watersheds cover an area ~80,000 km^2^, total freshwater discharge of ∼21 km^3^ yr^−1^, and drain about 40% of North Carolina’s and 10% of Virginia’s surface area. Because tidal exchange with the coastal Atlantic Ocean is restricted to three narrow inlets, the APS has a relatively long water residence time of ~1 yr^14^; this provides suspended algae (phytoplankton) and vascular plants ample time to assimilate nutrients, resulting in high productivity per unit nutrient input. These characteristics are key to the PS serving as a highly productive nursery, supporting ~80% of US mid-Atlantic commercial and recreationally caught finfish and shellfish species^11^. However, it also makes the system sensitive to nutrient-over enrichment, resultant eutrophication and nuisance algal blooms^12,15^. The long residence time also enables ample time for photochemical and/or microbial degradation of organic matter^16^.

### Monitoring and sampling

Biogeochemical and water quality data were obtained from the long-term ModMon monitoring programs in the NRE and western PS^10^. ModMon is a collaborative University - State of North Carolina (NC-Dept. of Environmental Quality-DEQ), and UNC-Chapel Hill Institute of Marine Sciences (IMS) program (http://paerllab.web.unc.edu/projects/modmon/), initiated in 1994. Sampling consisted of twice monthly visits to 11 mid-river stations along the estuarine portion of NRE (Fig. 2), including vertical profiles with collections of near-surface and near-bottom water for physical-chemical-biological parameters. Monthly samples were also collected at nine stations in the western PS as part of the ModMon program starting in 2000 (Fig. 2). Profiles of temperature, salinity, and dissolved oxygen were made at 0.5 m depth intervals using YSI 6600 multi-parameter water quality sondes (Yellow Springs Inc, Yellow Springs, Ohio). Sondes were calibrated prior to each sampling trip according to the YSI User’s Manual.

Samples for nutrient and organic matter concentrations and phytoplankton biomass were collected along the ModMon transect in the NRE from 1994 through 2018, and in the western PS from 2000 through 2018. Water samples were collected at the surface (0.2 m depth) and bottom (0.5 m above bottom) using a non-destructive diaphragm pump, dispensed into 4 L polyethylene bottles, and returned for processing within 4 h of collection to the laboratory at the UNC-CH Institute of Marine Sciences, Morehead City (IMS).

### Nutrient and carbon analyses

Nutrient measurements included: total dissolved nitrogen (TDN), nitrate plus nitrite (NO_x_ = NO_3_^−^ + NO_2_^−^), ammonium (NH_4_^+^), soluble reactive phosphate (SRP), dissolved organic carbon (DOC), dissolved inorganic carbon (DIC), particulate organic carbon (POC), and particulate nitrogen (PN). Dissolved inorganic nitrogen (DIN) was calculated as NO_3_^−^ + NO_2_^−^ + NH_4_^+^. Dissolved organic nitrogen (DON) was computed by difference as TDN – DIN. Details on sample preparation and processing are in^17^. Filtrates were analyzed for dissolved N forms and SRP with a Lachat/Zellweger Analytics QuickChem 8000 flow injection autoanalyzer using standard protocols (Lachat method numbers 31-107-04-1-C, 31-107-06-1-B, and 31-115-01-3-C, respectively)^17^. Particulate organic carbon (POC) and nitrogen (PON) were measured on seston collected on pre-combusted GF/F filters, analyzed by high-temperature combustion using a Costech ECS 4010 analyzer^18^. DIC and DOC were measured on a Shimadzu Total Organic Carbon Analyzer (TOC-5000A)^19^.

### Phytoplankton biomass

Chlorophyll *a* (Chl-*a*) was measured for near-surface and near-bottom samples by filtering 50 mL of NRE water onto GF/F filters. Filters were frozen at −20 °C and subsequently extracted using a tissue grinder in 90% acetone^17^. Chl-*a* of extracts was measured using the non-acidification method of Welschmeyer^20^, on a Turner Designs Trilogy fluorometer calibrated with pure Chl-*a* standards (Turner Designs, Sunnyvale, CA).

### Freshwater discharge, and material loading

Daily average Neuse River discharge was measured by the United States Geological Survey (USGS) at Fort Barnwell (USGS 02091814), and divided by 0.69 to account for ungaged downstream inputs^17^. Daily Neuse River loads of carbon and nutrient forms were estimated using Weighted Regressions on Time Discharge and Season (WRTDS)^21,22^, based on daily average discharge and concentrations measured by ModMon (or NC DEQ for total N and total P) at the head of the estuary (Fig. 2). Half-window widths of the tricube weight function for seasonality, time, and discharge were set to default values of 6 months, 7 years, and 2 natural log units, respectively^21^.

A long term record of precipitation events in the Neuse River basin at Kinston, NC was assembled from National Oceanic and Atmospheric Administration Cooperative Observer Network sites 314684 (1 September 1899 to 15 June 2017) and site 314689 (15 June 2017 to 5 December 2018). Data from 1 May 1919 to 29 November 1923 were not available. Precipitation events were defined as daily precipitation greater than 4.85 cm, the 99^th^ percentile of daily precipitation. Consecutive days of rainfall greater than 4.85 cm were considered the same event and assigned to the day the event began. Precipitation events were ascribed to tropical cyclones when a precipitation event was coincident with the passage of a tropical cyclone within 240 km (150 miles) of Kinston, NC as determined by 6 h storm advisories recorded in the National Hurricane Center’s HURDAT2 database. Quantile regressions were constructed for the 90^th^ and 50^th^ quantiles of cyclone related precipitation against time. 95% confidence intervals on slopes for the quantile regressions were determined by 1000 rounds of bootstrapping. A long term record (1 May 1930 to 8 December 2018) of high river flow events for the Neuse River was assembled from the USGS gage at Kinston, NC (USGS gage 02089500). High flow events were those during which the daily average flow was greater than 390 m^3^ s^−1^, the 99^th^ percentile of daily average flow. Consecutive days of flow greater than 390 m^3^ s^−1^ were considered the same event and summed from the day the event began to determine the total event discharge. Discharge events were ascribed to tropical cyclones when a discharge event followed within a seven-day period the passage of a tropical cyclone within 240 km (150 miles) of Kinston, NC. Discharge was regulated by Falls Lake dam from 1982 to the present.

## Results and Discussion

### Extreme rainfall associated with recent tropical cyclones

A conservative estimate of the probability of receiving two 2-percent annual exceedance level floods and one 0.8-percent annual exceedance level flood in a span of two decades can be calculated, assuming independence of these events. The probabilities of these flow levels not being exceeded are 0.98 and 0.992. The probability of three such events occurring in a twenty-year period is

$${\rm{P}}=(1\,-\,{0.98}^{20})\,\ast \,(1\,-\,{0.98}^{20})\,\ast \,(1\,-\,{0.992}^{20})=0.016$$

With less than a 2% chance of three such events occurring in a twenty-year period^23^, either North Carolina has been very unlucky, or the historical record used to define the storm statistics is no longer representative of the present climatic regime. This analysis suggests that the occurrence of three extreme floods resulting from high rainfall tropical cyclone events in the past 20 years is a consequence of the increased moisture carrying capacity of tropical cyclones due to the warming climate^4,6,24–26^.

While we do not offer a full attribution analysis, which may be conducted in a variety of ways including numerical modeling that replicates the events^27,28^, our observations are consistent with observations elsewhere and with predicted changes in a warming climate^2–6^. Moreover, rather than attributing a particular event to global warming, we should consider whether a warming climate made these events more likely, which our records suggest is the case for coastal NC. For example, increased precipitation in other US coastal areas subject to tropical cyclones (e.g., coastal Texas from Hurricane Harvey in 2017)^29–31^ and increased hurricane activity since 1970^30^ have been attributed to global warming. Factors potentially driving the increased precipitation include; (1) greater heat content of ocean waters, which not only fuels storm intensity but also increases precipitation^29^, (2) a decrease in tropical cyclone forward movement^32^ providing more opportunity for heavy precipitation over a particular area, (e.g. Harvey and Florence), (3) an observed poleward migration of tropical cyclones^33^, perhaps making coastal NC more vulnerable than in the past, and (4) an increase in tropical cyclone intensity in the satellite era^34^.

**Figure 3:**
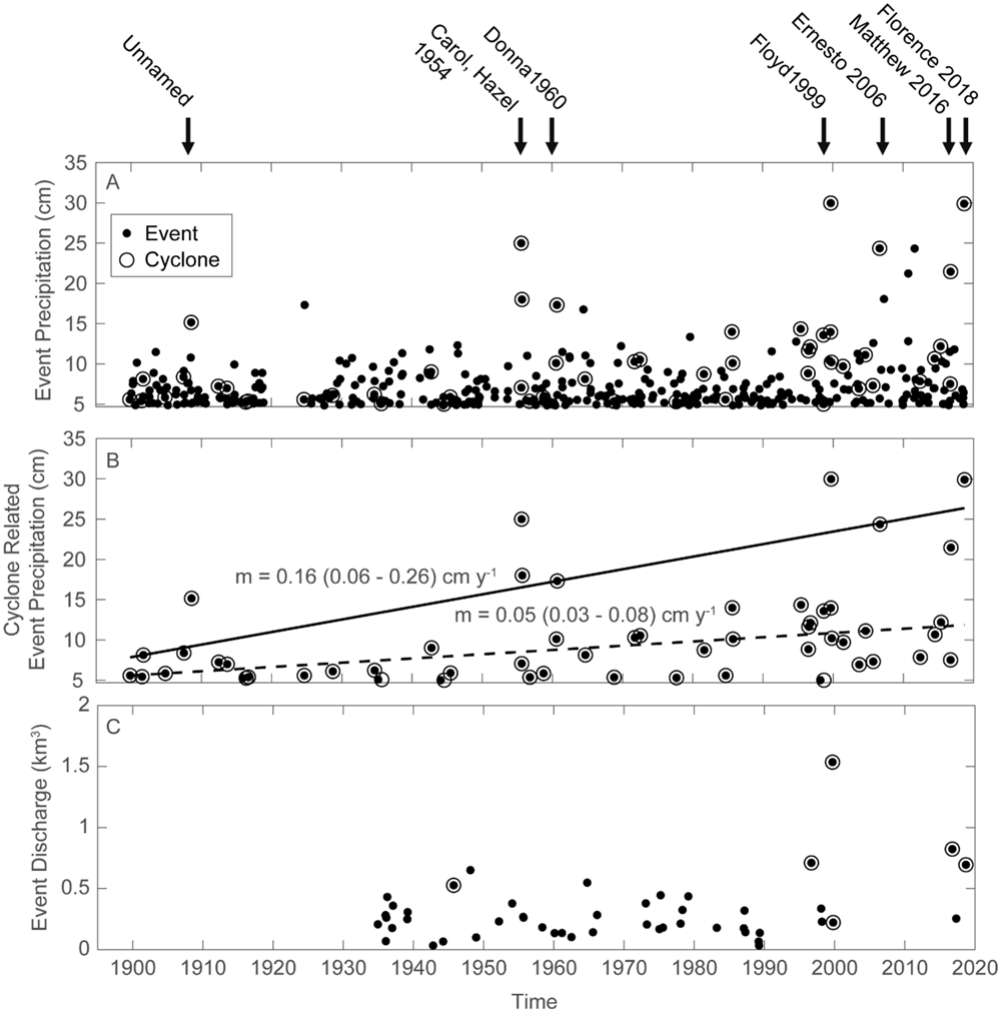
(**A**) Time series of high precipitation events at Kinston, NC from 1899 to 2018. (**B**) 50^th^ and 90^th^ quantile regressions of cyclone related precipitation against time. 95% confidence intervals of slopes, m, shown in parentheses. (**C**) Total volumetric discharge for high flow events at Kinston, NC (USGS gage 02089500) from 1 May 1930 to 8 December 2018.

Fortunately, North Carolina has a well-kept continuous record of tropical cyclone landfalls and associated rainfall since 1898, which we investigated in order to further test the hypothesis that we have recently entered a regime shift of increased extreme rainfall and associated flooding. Three periods of elevated cyclone activity were noted; the first in the early 1900’s (~1910), then during the 1950’s and most recently since the mid-1990’s (Fig. 3A). However, six of the seven highest precipitation events, four of the six due to tropical cyclones, have occurred in the past 20 years. Both the median and 90% quantile of precipitation from cyclone-related extreme precipitation events have increased significantly over the past century (Fig. 3B), and a more rapid increase in the 90% quantile reflects the recent occurrence of those six very high precipitation events. In addition, these events have been accompanied by record freshwater discharge to the NRE (Fig. 3C). The rank of the total event discharge in Fig. 3 does not necessarily correspond to its rank in peak flow. For example, total event discharge following Hurricanes Floyd (1999) and Florence (2018) were significantly higher than following Hurricane Matthew (2018) which had the highest instantaneous peak flows and flood stage. Part of this result was due to continued heavy rainfall following hurricanes Floyd (including Hurricane Irene) and Florence, and following Florence was also due to retention and slow release of flood waters through Falls Lake dam upstream from the NRE.

### Biogeochemical and water quality impacts

**Figure 4:**
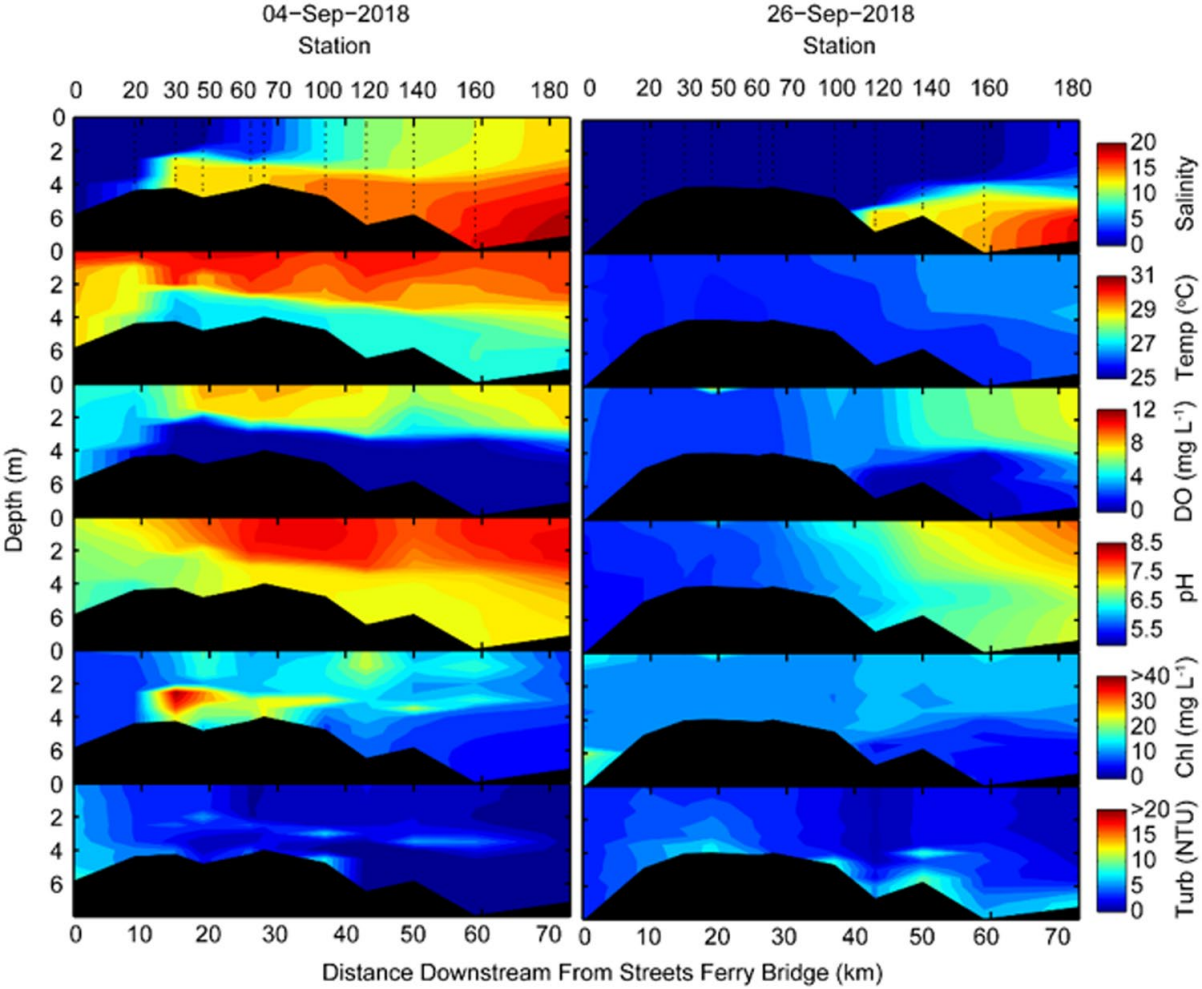
Downstream and vertical gradients of key water quality parameters (salinity, temperature, dissolved oxygen, pH, and turbidity) in the Neuse River Estuary, NC before and after Hurricane Florence, which made landfall on 14 September, 2018. The downstream transect covers from the historic upstream extent of salt water instrusion downstream to Pamlico Sound.

**Figure 5:**
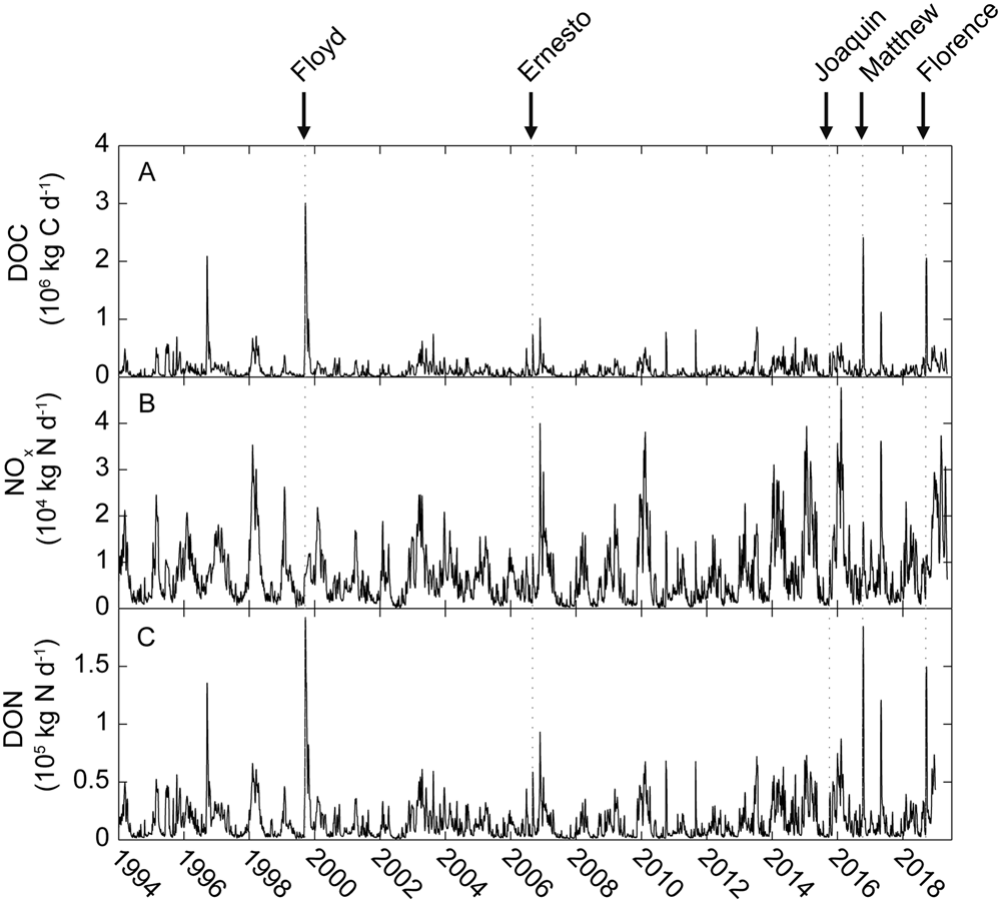
Time series of loads of major forms of organic carbon (Dissolved organic carbon-DOC) and nitrogen (NO_x_, Dissolved organic nitrogen-DON) to the Neuse River Estuary.

**Figure 6:**
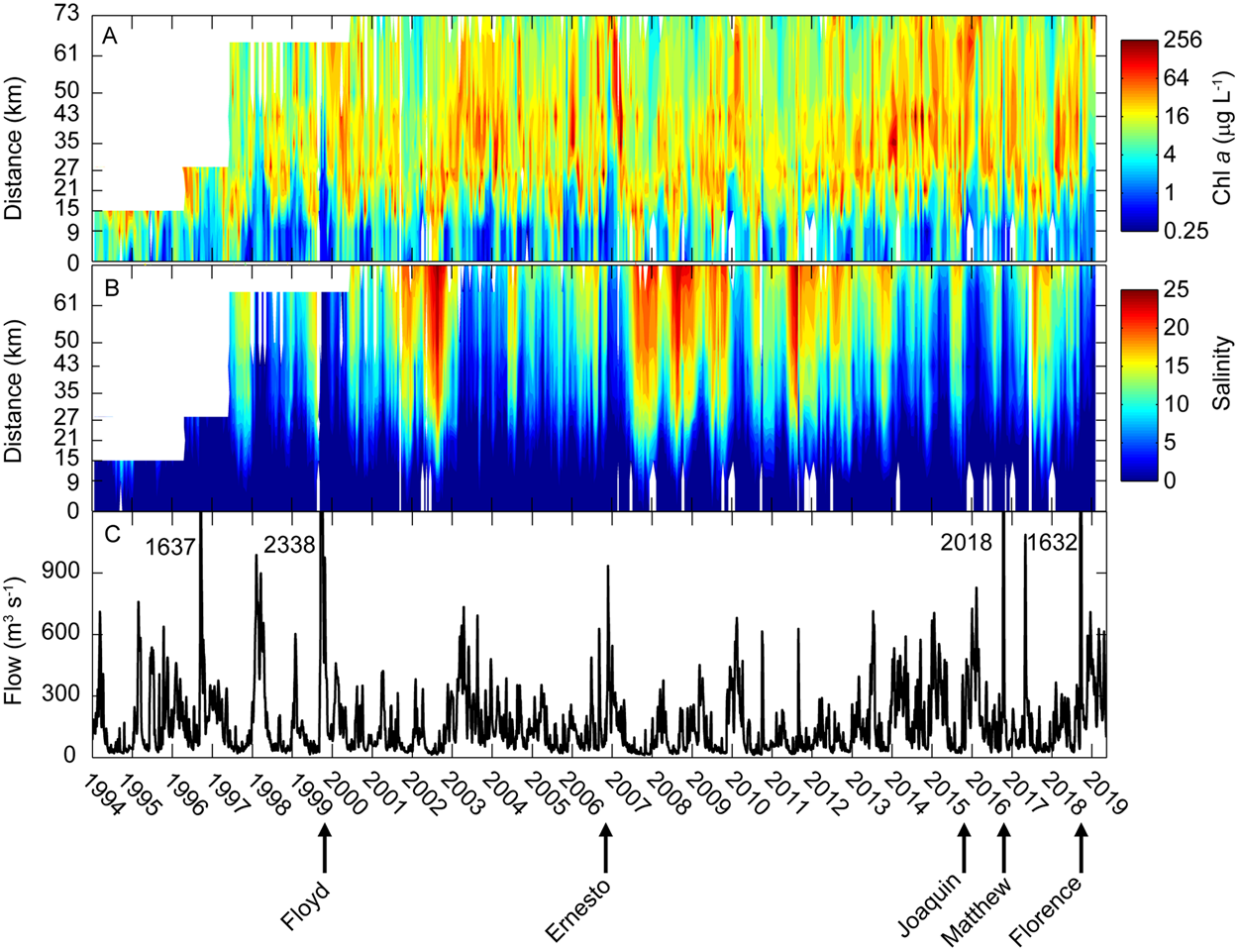
Time series of river flow at Streets Ferry Bridge and contour plots showing the time series of downstream distributions of salinity and chlorophyll *a* in the Neuse River Estuary.

The most recent extreme precipitation events each have delivered up to 100 cm of rainfall to coastal watersheds; often accounting for 30–45% of the average annual rainfall^8^. The floodwaters resulting from Hurricane Florence (~14 September, 2018) completely “freshened” the entire NRE, leaving only an oxygen poor salt wedge downstream at its mouth, near the entrance to Pamlico Sound (Fig. 4). The floodwaters contained extremely high loads of organic matter^35^, dominated by dissolved organic carbon (DOC) and dissolved organic nitrogen (DON) as well as nutrients, specifically the growth-limiting nutrient nitrogen^36^, shown here as NO_x_, (Fig. 5). In addition to depressing salinity throughout the NRE, the floodwater nutrient load fueled phytoplankton production and subsequent algal blooms (as chlorophyll *a*), which were promoted after the flow rates (and hence flushing) decreased, enabling phytoplankton biomass to build up in the estuary (Fig. 6). Phytoplankton blooms and associated hypoxia often continued for days to weeks after a storm had passed.

**Figure 7:**
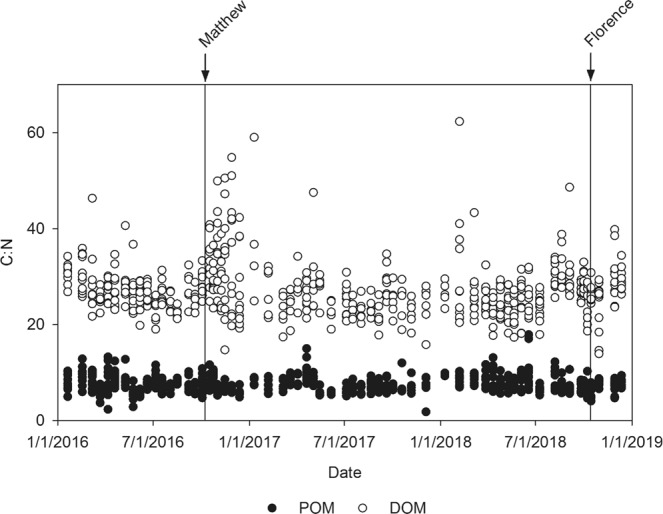
Time series of molar C:N ratios for the dissolved and particulate organic matter fractions in surface waters of the Neuse River Estuary from 2016 to 2018. Each date shows data from down the entire estuary.

Analysis of C:N content of the dissolved and particulate organic matter entering the NRE (Fig. 7) indicated that a vast proportion of the DOM have quite high C:N ratios (>20), consistent with terrigenously-derived sources that were evident from satellite images of the NC coast (Fig. 1). Degradation of this terrigenous DOM by a combination of sunlight and bacteria may have kept the Pamlico Sound as a net CO_2_ source to the atmosphere in the weeks following Hurricane Matthew^35^. In contrast, POM had C:N ratios <10 (Fig. 7), indicating authochtonous sources, most likely phytoplankton production, in this eutrophic estuary. Sustained primary production has the potential to modulate CO_2_ dynamics by creating a CO_2_ sink^18,35^.

Thus, evidence is accumulating that we may also be seeing changes to the “system state” of coastal waters in terms of their ability to capture or release CO_2_^37,38^. Such changes caused by an increased frequency of extreme storm events are ostensibly reorganizing coastal carbon cycles^38^. For example, flood waters reaching the inner shelf of the Gulf of Mexico have resulted in extensive degradation of terrestrial organic matter and the return of that carbon as CO_2_ to the atmosphere^38^. Further, the biogeochemistry of coastal waters is inextricably linked to their water quality^9,15^. Floodwater-associated nutrients have been shown to promote harmful algal blooms (HABs) in these systems^39^. Floodwaters contain contaminants and runoff from urban and agricultural land use^13^, and due to the high organic load, flood waters are often hypoxic when they enter an estuary, which was evident in the %DO values < 40% in the upper NRE following each storm (Fig. 4). Additionally, high freshwater inflows reduce vertical mixing and “trap” denser salt water causing extensive hypoxia in bottom waters extending throughout the estuary (Fig. 4). These hypoxic events can last weeks to months and provide the ingredients for massive finfish and shellfish kills, as well as an abrupt increase in fish disease^36,40^

Losses suffered by coastal communities from these events can be catastrophic. All of the aforementioned biogeochemical effects have severe economic and societal implications for fisheries, tourism, and real-estate, and have raised concerns about coastal resiliency and sustainability. In North Carolina alone, Hurricane Floyd in 1999 caused fisheries losses of US$6 million and overall economic (tourism, property and business damage and losses, agriculture and silviculture) amounted to US$2 billion^41^.

While the hydrologic, nutrient and carbon inputs attributable to Florence (Sept.–Nov. 2018) are yet to be fully tallied, the rainfall associated with this event was roughly equivalent to Matthew, in 2016. Like Floyd and Matthew, Florence’s floodwaters led to “freshening” and expanding hypoxic zones in the APS system (Fig. 4), as well as massive pulses of carbon overflowing from the APS into coastal waters, as viewed from space (Fig. 1), with effects that can linger for months after a storm^35^.

## Conclusions

Considering these extreme precipitation events and their hydrologic and biogeochemical consequences in totality, it is clear that they are unparalleled in the past 120+ years of recorded tropical cyclones in coastal North Carolina (Fig. 3). The potential exists for receiving waters globally to undergo unprecedented perturbations to nutrient and carbon cycling, fisheries habitat and sustainability due to increasing frequency of extreme precipitation events; all of which are still to be determined. With roughly 40% of the world’s population within 100 km of the coast, development inland, as well as along the coastline, will exacerbate the perturbations caused by this type of regime shift^42^. We stress that stakeholders, state and federal governments need to better prepare for the acute as well as cumulative water quality, fisheries resource and overall socio-economic effects of this recently-documented rise in catastrophic flooding associated with elevated tropical storm activity.
